# Duodenal mucosal risk markers in patients with familial adenomatous polyposis: effects of celecoxib/ursodeoxycholic acid co-treatment and comparison with patient controls

**DOI:** 10.1186/1750-1172-8-181

**Published:** 2013-11-19

**Authors:** Bjorn WH van Heumen, Hennie MJ Roelofs, René HM te Morsche, Fokko M Nagengast, Wilbert HM Peters

**Affiliations:** 1 Department of Gastroenterology, Radboud University Nijmegen Medical Centre, PO Box 9101, 6500 HB Nijmegen, The Netherlands

**Keywords:** Familial adenomatous polyposis (FAP), Duodenal mucosa, Risk markers, Celecoxib, Ursodeoxycholic acid, Duodenal adenomatosis, Glutathione S-transferase Alpha, Caspase-3

## Abstract

**Background:**

Familial adenomatous polyposis (FAP) is a disease characterized by the development of hundreds to thousands of adenomatous polyps in the colorectum early in life. Virtually all patients with FAP will develop colorectal cancer before the age of 40 to 50 years, unless prophylactic colectomy is performed, which significantly improves their prognosis. The mortality pattern has changed and duodenal cancer now is one of the main cancer-related causes of death in these patients. Practically all patients with FAP develop premalignant duodenal adenomas, which may develop to duodenal cancer in approximately 3-7% of patients. Duodenal cancer in patients with FAP has a poor prognosis. The clinical challenge is to identify patients at high-risk for duodenal carcinoma. Chemoprevention would be desirable to avoid duodenectomy. The main goal of this study is to identify risk markers in normal duodenal mucosa of patients with FAP, that could help identify patients at increased risk for malignant transformation.

**Methods:**

Messenger RNA (mRNA) levels of glutathione S-transferase A1 (GSTA1), glutathione S-transferase P1 (GSTP1), KIAA1199, E-cadherin, peroxisome proliferative activated receptor δ (PPARδ), caspase-3, cyclin D1, β-catenin, and cyclooxygenase-2 (COX-2) were measured in duodenal mucosa, using the QuantiGene 2.0 Plex assay. Levels in normal appearing mucosa of patients with FAP (n = 37) were compared with levels in non-FAP patient controls (n = 16). In addition, levels before and after treatment with either celecoxib & ursodeoxycholic acid (UDCA, n = 14) or celecoxib & placebo (n = 13) were evaluated in patients with FAP.

**Results:**

mRNA levels of glutathione S-transferase A1 (28.16% vs. 38.24%, p = 0.008) and caspase-3 (3.30% vs. 5.31%, p = 0.001) were significantly lower in patients with FAP vs. non-FAP patient controls, respectively. COX-2 mRNA levels in normal duodenal mucosa of patients with FAP were found to be unexpectedly low. None of the potential risk markers was influenced by celecoxib or celecoxib & UDCA.

**Conclusions:**

Protection against toxins and carcinogens (GSTA1) and apoptosis (caspase-3) is low in patients with FAP, which could contribute to increased susceptibility for malignant transformation of duodenal mucosa.

**Trial registration:**

http://ClinicalTrials.gov number NCT00808743

## Background

Familial adenomatous polyposis (FAP), characterized by the development of numerous premalignant colorectal adenomatous polyps, is caused by a germline mutation in the tumor suppressor *adenomatous polyposis coli* (APC) gene [1]. In the past decades, preventing development of colorectal cancer by prophylactic colectomy substantially improved prognosis in patients with FAP [2]. As a result, the mortality pattern has changed and duodenal cancer now is the leading cancer-related cause of death [3,4]. Lifetime risk of duodenal adenomas approaches 100% in patients with FAP [5], and approximately 3-7% of patients eventually develop duodenal cancer [6,7]. As duodenal cancer in patients with FAP has been associated with a poor prognosis [8,9], the clinical challenge is to identify patients with high-risk duodenal adenomas and intervene before progression to cancer occurs. Identification of early risk markers in normal duodenal epithelium could help identify patients at increased risk for malignant transformation.

Potentially useful biomarkers can be expected in cellular pathways that are linked to the affected *APC* gene and its translational product. APC is a multifunctional protein involved in regulation of cell proliferation, cell migration, cell adhesion, cytoskeletal reorganisation, and chromosomal stability [10]. The role of APC in intestinal carcinogenesis is attributed largely to the Wnt signaling pathway, but disruption of intercellular adhesion and stability of the cytoskeleton seems to be involved as well [11]. Loss of functional APC results in accumulation of cytosolic β-catenin and subsequent translocation to the nucleus, where β-catenin associates with members of the T cell factor (Tcf) and lymphoid enhancer factor family (lef) [11]. The β-catenin/Tcf complex activates several transcriptional targets, including the G1/S-regulating cyclin D1 [12] and the peroxisome proliferator-activated receptor δ (PPARδ) [13]. In addition, β-catenin also functions as an essential component of epithelial intercellular adherens junctions, where it links the cytoplasmic tail of E-cadherin to α-catenin which binds actin and actin-associated proteins of the microtubule cytoskeleton [14]. KIAA1199 was recently described as a novel target of the Wnt signaling pathway, in both colon and gastric carcinogenesis [15,16]. While disruptions in the Wnt signaling pathway are involved in tumor initiation [17], abnormal expression of cyclooxygenase-2 (COX-2) observed in the majority of adenomas and carcinomas, is thought to play a crucial role in tumor progression by increasing the levels of prostaglandin E2 (PGE2) [18]. Overexpression of COX-2 is linked to reduced apoptosis, enhanced cell growth, tumor angiogenesis, and tissue invasion and metastasis [19]. The involvement of the COX-2/PGE2 pathway may also explain the observed chemopreventive effects of COX inhibiting non-steroidal anti-inflammatory drugs (NSAIDs), decreasing the occurrence of sporadic colorectal adenomas [20,21]. Treatment with the COX-2 selective inhibitor celecoxib was found associated with regression of colorectal adenomas in patients with FAP [22]. Significant reduction in duodenal polyp density in patients with FAP with clinically significant disease was achieved with high-dose celecoxib [23], a finding we recently confirmed [24]. Combining celecoxib with other potentially effective drugs could reveal more effective strategies. Based on several preclinical and clinical studies, ursodeoxycholic acid (UDCA) was a candidate drug [25-29]. However, combining celecoxib with UDCA was found ineffective in reducing duodenal polyp density in patients with FAP [24].

The clustering of adenomas around the ampulla of Vater suggests that cytotoxic bile plays a role in duodenal adenomatosis in patients with FAP [30]. Detoxification enzymes, such as glutathione S-transferases (GSTs), protect the gastrointestinal mucosa against exogenous and endogenous toxic, mutagenic or carcinogenic compounds by catalyzing the conjugation with glutathione [31]. In patients with FAP, a significantly lower GST activity was observed in colonic mucosa as compared to healthy controls [32]. Distorted expression levels of detoxification enzymes in the duodenum, could reduce functional activity and modulate individual susceptibility for development of duodenal adenomas and carcinomas in patients with FAP.

Aim of the present study was to gain further insight into the cellular targets of potential chemopreventive treatment for duodenal adenomatosis in patients with FAP, as well as to define epithelial risk markers of malignant transformation. We determined messenger RNA (mRNA) expression levels of potential risk markers in duodenal epithelium of patients with FAP in comparison to non-FAP patient controls. Furthermore, we investigated the effects of treatment with celecoxib or celecoxib & UDCA co-treatment on mRNA expression of these selected biomarkers.

## Methods

### Study participants

The study population consists of patients with FAP and non-FAP patient controls. The patients with FAP, recruited at the Radboud University Nijmegen Medical Centre (RUNMC), Academic Medical Centre Amsterdam (AMC), Erasmus Medical Centre Rotterdam (EMC), University Medical Centre Groningen (UMCG), and Leiden University Medical Centre (LUMC), participated in a double-blind randomized clinical trial (http://ClinicalTrials.govnumberNCT00808743), which is described in detail elsewhere [24]. In short, after completion of pre-intervention gastroduodenoscopy, patients with FAP were randomly assigned to one of two treatment groups. Patients in group A received celecoxib (Celebrex, Pfizer, New York, NY, USA) 400 mg twice daily for 6 months in combination with UDCA (20-30 mg/kg body weight daily; Ursofalk, Dr Falk Pharma, Freiburg, Germany). Patients in group B received celecoxib 400 mg twice daily in combination with an UDCA identical-appearing placebo (Dr Falk Pharma). The diagnosis FAP was established either clinically, by the presence of >100 colorectal polyps, or genetically, by the presence of adenomatous polyposis coli (*APC*) gene mutations. Eligible patients were between 18 and 70 years of age, capable of informed consent, had Spigelman stages II or III at last surveillance gastroduodenoscopy, and had no history of surgical duodenal resection.

Patient controls were recruited at the Department of Gastroenterology, RUNMC. All patients aged 18 to 70 years and scheduled for diagnostic gastroduodenoscopy because of dyspepsia, or follow-up after previous diagnosis of upper gastrointestinal dysplasia or Barrett’s metaplasia, were selected. With permission from the referring physician, patients received a study information leaflet and an informed consent form by post. They were contacted by telephone one week before the planned gastroduodenoscopy to inquire if additional information was necessary, whether they were willing to participate, and if so, if any of the exclusion criteria were applicable. Exclusion criteria were: use of NSAIDs or UDCA for >1 week during 3 months prior to study entry, history of inflammatory bowel disease, upper gastrointestinal cancer, upper gastrointestinal surgery, celiac disease, pregnancy, or lactation. Informed consent was obtained prior to gastroduodenoscopy. Patient controls were compensated for participation with €100.

From all participants, four random biopsies of normal appearing mucosa were taken in the D2 segment of the duodenum, as well as four random biopsies in the D3/D4 segment. An Olympus Endojaw FB-232U with open forceps diameter 9 mm, or a Boston Scientific Radial Jaw 3 with open forceps diameter 8 mm (Boston Scientific, Natick, MA, USA) was used. Biopsies were snap frozen in liquid nitrogen and stored at -80°C for mRNA analyses. Patients with FAP underwent gastroduodenoscopy twice: at baseline and after the intervention period.

The present study was conducted according to ICH Good Clinical Practice and complied with the principles of the amended Declaration of Helsinki and Dutch legislation. Ethical approval was obtained at the initiating centre Radboud University Nijmegen Medical Centre (RUNMC; number 2008/148; CCMO number NL23569.091.08). All study participants provided written informed consent. Disclosure of randomization was performed by the Department of Clinical Pharmacy, RUNMC, on December 10th 2012, after all tissue assessments and analyses were completed.

### Isolation of RNA from biopsies and quantification of duodenal mRNA levels

One biopsy of each location was weighed and taken up in 200 μL TRIzol (Life Technologies, Pailey, UK). Tissue was homogenized by 10 strokes with a Teflon pestle and after homogenization another 600 μL TRIzol reagent was added. Total RNA was extracted according to the manufacturer’s instructions (Life Technologies) with a slight modification as follows: prior to precipitating the RNA with iosopropyl alcohol, 7.5 μg RNAse-free glycogen was added as a carrier to the aqueous phase. Approximately 1 μg of total purified RNA was used for the QuantiGene 2.0 Plex assay (Affymetrix, Santa Clara, CA, USA).

RNA was incubated with Luminex beads and capture probes according to the protocol of the manufacturer. Target-specific probe sets for beta-2-microglobulin (β2M, NM_004048), glutathione S-transferase A1 (GSTA1, NM_145740), glutathione S-transferase P1 (GSTP1, NM_000852), KIAA1199 (NM_018689), E-cadherin type 1 (CDH1, NM_004360), peroxisome proliferative activated receptor delta (PPARD, NM_006238), caspase-3 (CASP3, NM_004346), cyclin D1 (CCND1, NM_053056), beta-catenin-1 (CTNNB1, NM_001904) and cyclooxygenase-2 (COX-2, NM_000963) were developed by Affymetrix. Signals of cascade amplification of the fluorescent microspheres were detected by the Biorad Luminex100 Bio-Plex system using the Bio-Plex Manager 4.1 software (Bio-Rad Laboratories, Hercules, CA). The mRNA expression level of the housekeeping gene β2M in each sample was used for normalization. mRNA levels were expressed as percentage relative to the levels of β2M, which were set at 100%. The mean of the mRNA expression levels at the D2 and D3/D4 locations was used in the analyses.

### Statistical analysis

Baseline characteristics were expressed as percentage or medians with range where appropriate. Outcome variables were expressed as group medians with 25 and 75 percentiles. Differences on discrete variables were examined using Chi-square test, or Fisher’s exact test when appropriate. Continuous variables were considered to be not normally distributed. Differences on continuous variables of baseline characteristics and outcome measurements between groups, including measurements in patients with FAP at baseline versus non-FAP patient controls, and measurements in FAP patients treated with celecoxib & UDCA versus patients treated with celecoxib & placebo, were tested using Mann–Whitney U test. Differences within study groups, comparing pre- and post-intervention measurements in patients with FAP, were examined using Wilcoxon Signed Rank test. For baseline characteristics, a p-value of <0.05 (2-sided) was considered statistically significant. Since each analysis of the outcome measurement comprised a set of nine mRNA expression levels, a correction for multiple testing was applied. In each of these analyses, a p-value of <0.01 was considered statistically significant. Statistical analysis was performed using SPSS statistical software version 21 (IBM SPSS, Chicago, IL, USA).

## Results

### Patient characteristics

Patient characteristics of patients with FAP and non-FAP patient controls are depicted in Table 1. Thirty-seven patients with FAP were randomized: 19 patients received celecoxib & UDCA (group A) and 18 patients received celecoxib & placebo (group B). Nine patients with FAP (24.3%; group A, n = 5; group B, n = 4) discontinued intervention prior to post-intervention gastroduodenoscopy at 6 months. In one patient in group B, post-intervention biopsies could not be processed and for this patient only pre-intervention measurements were included in the analyses.

**Table 1:** Baseline characteristics of the study population consisting of patients with familial adenomatous polyposis (FAP) and non-FAP patient controls

	**Overall non-FAP group**	**Overall FAP group**	**p-value**	**FAP group A: Celecoxib & UDCA**	**FAP group B: Celecoxib & placebo**	**p-value**
Number of patients	16	37		19	18	
Age at study entry, median/range (yr)	53/23-67	42/22-67	0.092*	42/22-67	41/27-64	0.964*
Sex (n,%)			0.241†			0.618†
Male	5 (31)	18 (49)		10 (53)	8 (44)	
Female	11 (69)	19 (51)		9 (47)	10 (56)	
Body Mass Index, median/range (kg/m^2^)	26.1/19.4-44.2	25.6/18.8-34.5	0.779*	26.0/19.2-34.5	25.6/18.8-33.1	0.408*
Spigelman score at last surveillance before entry						0.985†
II		19 (51)‡		10 (53)	9 (50)‡	
III		17 (46)‡		9 (47)	8 (44)‡	

*The p-value was calculated by the Mann–Whitney U test.

†The p-value was calculated by the Chi-square test.

‡In 1 case data on Spigelman score at last surveillance gastroduodenoscopy before study entry was missing.

*Abbreviations*: FAP, familial adenomatous polyposis; UDCA, ursodeoxycholic acid.

Seventeen non-FAP patient controls underwent gastroduodenoscopy as prescribed by the study protocol. One patient control was excluded from analyses, due to diagnosis of celiac disease based on histopathological examination of the biopsies, and 16 non-FAP patient controls were included in the analyses. Indications for gastroduodenoscopy were dyspepsia (n = 10), iron deficiency anemia (n = 1), follow-up after previous dysplasia or metaplasia in the upper gastrointestinal tract (n = 4), and follow-up after Helicobacter pylori eradication (n = 1).

### Patients with FAP vs. non-FAP patient controls

Median mRNA levels of selected genes in endoscopically normal appearing mucosa of patients with FAP before clinical intervention (n = 37) were compared with the levels in non-FAP patient controls (n = 16). Results are included in Tables 2 and 3.

**Table 2:** Duodenal mRNA levels of selected genes

			**n**	**Messenger RNA**								
				**KIAA1199**	**COX-2**	**CDH1**	**PPARD**	**GSTA1**	**CASP3**	**CCND1**	**CTNNB1**	**GSTP1**
Patient controls			16	0.04 (0.03-0.06)	0.05 (0.04-0.07)	16.43 (11.54-22.14)	0.73 (0.52-1.12)	38.24 (27.25-51.76)	5.31 (4.14-6.77)	4.20 (2.71-5.95)	26.90 (21.88-36.03)	9.69 (6.37-15.60)
Patients with FAP	Total	Pre-intervention	37	0.05 (0.03-0.24)	0.04 (0.03-0.05)	13.36 (10.82-15.73)	0.71 (0.48-0.95)	28.16 (21.62-37.90)	3.30 (2.46-4.68)	3.72 (3.03-4.69)	24.84 (19.02-28.45)	7.64 (5.70-10.71)
	Group A: Celecoxib & UDCA	Pre-intervention	19	0.04 (0.02-0.28)	0.04 (0.03-0.06)	13.54 (10.90-18.51)	0.74 (0.54-1.05)	30.30 (23.16-36.54)	3.30 (2.47-5.21)	3.96 (2.63-4.72)	25.89 (19.80-31.89)	7.59 (5.78-11.07)
		Post-intervention	14	0.04 (0.02-0.23)	0.04 (0.03-0.05)	13.72 (10.46-15.21)	0.63 (0.50-1.28)	27.13 (21.28-33.30)	3.20 (2.88-4.11)	3.50 (2.81-5.24)	24.05 (20.96-30.27)	7.36 (6.22-10.47)
	Group B: Celecoxib & placebo	Pre-intervention	18	0.09 (0.03-0.22)	0.03 (0.02-0.05)	11.66 (10.28-15.42)	0.63 (0.45-0.87)	27.96 (19.84-39.44)	3.30 (2.42-4.43)	3.63 (3.18-4.63)	21.60 (18.63-27.89)	7.73 (5.46-10.27)
		Post-intervention	13	0.24 (0.05-0.84)	0.05 (0.03-0.08)	14.08 (10.79-16.87)	0.78 (0.58-1.20)	31.74 (21.02-47.12)	2.94 (2.58-4.83)	4.56 (3.09-5.56)	25.26 (19.93-33.62)	10.82 (7.44-16.18)

*Abbreviations*: FAP, familial adenomatous polyposis; UDCA, ursodeoxycholic acid; β2M, beta-2-microglobulin; COX-2, cyclooxygenase-2; CDH1, E-cadherin type 1; PPARD, peroxisome proliferative activated receptor delta; GSTA1, glutathione S-transferase A1; CASP3, caspase 3; CCND1, cyclin D1; CTNNB1, beta-catenin-1; GSTP1, glutathione S-transferase P1.

Levels are expressed as median percentage relative to the mRNA level of the housekeeping gene β2M, set at 100%. Values are expressed as mean with 25 and 75 percentiles. For p-values of statistical analyses comparing mRNA levels of selected genes, see Table 3.

**Table 3:** P-values of statistical analyses comparing duodenal mRNA levels of selected genes

		**Statistical test**	**P-values**								
			**KIAA1199**	**COX-2**	**CDH1**	**PPARD**	**GSTA1**	**CASP3**	**CCND1**	**CTNNB1**	**GSTP1**
FAP pre-intervention vs. controls		Mann–Whitney U	0.253	0.021	0.060	0.438	0.008*	0.001*	0.373	0.269	0.104
FAP: pre- vs. post-intervention	Group A: celecoxib & UDCA	Wilcoxon Signed Rank	0.730	0.826	0.048	0.778	0.272	0.074	0.730	0.272	0.433
	Group B: celecoxib & placebo	Wilcoxon Signed Rank	0.046	0.345	0.064	0.279	0.173	0.600	0.075	0.101	0.013
	Group A vs. B: median of differences	Mann–Whitney U	0.048	0.720	0.006*	0.583	0.048	0.128	0.259	0.043	0.019

*Statistically significant with correction for multiple testing applied (p < 0.01).

*Abbreviations*: FAP, familial adenomatous polyposis; UDCA, ursodeoxycholic acid; COX-2, cyclooxygenase-2; CDH1, E-cadherin type 1; PPARD, peroxisome proliferative activated receptor delta; GSTA1, glutathione S-transferase A1; CASP3, caspase 3; CCND1, cyclin D1; CTNNB1, beta-catenin-1; GSTP1, glutathione S-transferase P1.

mRNA levels of GSTA1 and caspase-3 were significantly lower in patients with FAP when compared to levels in non-FAP patient controls (GSTA1: 28.16% [25-75%: 21.62%-37.90%] vs. 38.24% [27.25%-51.76%]; Mann–Whitney U, p = 0.008); caspase-3: 3.30% [2.46%-4.68%] vs. 5.31% [4.14%-6.77%]; Mann–Whitney U, p = 0.001). No statistically significant difference in median duodenal mRNA levels between patients with FAP and non-FAP patient controls were found for GSTP1, KIAA1199, E-cadherin-1, PPARδ, cyclin D1, β-catenin-1, and COX-2.

### Patients with FAP pre- vs. post-intervention

Pre- and post-intervention duodenal mRNA levels of selected genes were evaluated in patients with FAP, either treated with celecoxib & UDCA (group A, n = 14) or celecoxib & placebo (group B, n = 13). Results are shown in Tables 2 &3.

Comparison of median pre-intervention mRNA levels of the selected genes indicate that no differences existed at baseline between patients randomly assigned to group A vs. patients randomly assigned to group B (Mann–Whitney U, p > 0.05).

The only difference in effect when comparing both intervention groups was found for E-cadherin type 1 (Mann–Whitney U, p = 0.006). However, when evaluating the effects within each treatment group, no statistically significant change in mRNA level after treatment was observed, given the correction for multiple testing (group A: median difference = -1.35% [25-75%: -5.00%-0.68%], Wilcoxon Signed Rank, p = 0.048; group B: median difference = 1.76% [-0.61%-5.00%], Wilcoxon Signed Rank, p = 0.064). For any of the other mRNA levels, the interventions were found to have no statistically significant effects.

## Discussion

In the present study, expression of potential risk markers for malignant transformation were assessed by comparing their mRNA levels in normal appearing duodenal mucosa of patients with FAP with levels in non-FAP patient controls. Two important differences were revealed: duodenal mRNA levels of GSTA1 and caspase-3 were significantly lower in patients with FAP as compared to non-FAP patient controls. Lower duodenal levels of the detoxification enzyme GSTA1 could point at a lower capacity to detoxify toxins and carcinogens, with subsequent increased susceptibility for malignant degeneration [31]. Previously, we reported a significantly lower GST enzyme activity in colonic mucosa of patients with FAP, as compared to healthy controls [32], but surprisingly, no differences were found in duodenal mucosa of patients with FAP compared to patient controls [33]. However, the sample size in this study was low (n = 18), and GSTA1 and GSTA2 were simultaneously measured at the protein level [33]. In contrast, in the present study GSTA1 was selectively measured at mRNA level in 37 patients. GSTP1 levels were found to be similar in duodenum of patients with FAP and patient controls, which is in accordance with our previous data [33].

The lower level of caspase-3 found in patients with FAP, can also be considered as risk marker, as it suggests a decrease in apoptosis, with subsequent increased survival of cells with damaged DNA, prone for malignant degeneration. In a recent study, we were unable to detect apoptotic cells by immunohistochemistry in the normal duodenum of patients with FAP [24], which is consistent with our current finding using mRNA analysis.

Multiple lines of evidence, including results from *in vitro* studies, animal studies, as well as clinical studies, indicate that inhibition of COX-2 expression, at least in part, accounts for the anti-proliferative activity of celecoxib [34]. By using immunohistochemistry, COX-2 overexpression was reported in oesophageal [35], gastric [36], colorectal [37], and small intestinal cancer [38], as well as in normal duodenal mucosa of patients with FAP [39,40]. Moreover, also by immunohistochemistry, COX-2 levels in normal duodenal mucosa of patients with FAP were reported to be as high as levels in duodenal adenomas or carcinomas, and even higher than levels in normal colonic mucosa [40]. These findings are in sharp contrast with our results from the mRNA analysis. Although we did find high levels of COX-2 mRNA in normal colonic mucosa (Roelofs *et al*., unpublished results), hardly any mRNA expression was detected in normal duodenal mucosa of either patients with FAP or non-FAP patient controls. Similar results using qPCR analysis to evaluate mRNA expression were previously reported for small intestinal mucosa of non-FAP individuals [38]. These low levels of COX-2 mRNA in duodenal mucosa, in contrast to previous reports using immunohistochemistry, suggest that COX-2 is of minor importance in the initial processes of duodenal tumorigenesis in patients with FAP. Futhermore, the results cast doubt on the specificity of COX-2 detection by immunohistochemical staining.

Loss of functional APC in patients with FAP results in accumulation of cytosolic β-catenin and subsequent translocation to the nucleus [11], where the β-catenin/Tcf complex activates cyclin D1 [12] and PPARδ [13]. Our comparison of mRNA levels of cyclin D1 and PPARδ showed similar values in FAP and non-FAP duodenal mucosa (p = 0.37 and p = 0.44, respectively). Using immunohistochemistry, expression levels of β-catenin and E-cadherin were found to be lower in normal colon mucosa of patients with FAP as compared to non-FAP controls [41]. In addition, we previously described lower extracellular E-cadherin but higher cytoplasmic β-catenin expression in normal duodenal mucosa of patients with FAP, as compared to non-FAP controls [42]. In the current study, analysis of mRNA levels of β-catenin and E-cadherin markers in normal duodenal mucosa of patients with FAP and non-FAP patient controls could not confirm the previous immunohistochemical findings.

KIAA1199 was reported as a novel target of the Wnt signaling pathway and a putative marker for colorectal and gastic carcinogenic transformation [15,16]. We assessed KIAA1199 mRNA levels to investigate whether this marker is also expressed in the normal duodenal mucosa of patients with FAP with Spigelman grade II or III adenomatosis, consequently being at substantially increased risk of carcinoma development. However, duodenal KIAA1199 levels in normal mucosa of patients with FAP as well as in non-FAP controls were low and comparison did not reveal any difference. This could reflect the physiologic levels of KIAA1199 present at the proliferating crypt basis in the duodenal epithelium, as previously detected in the colonic epithelial crypts [15]. Analysis of KIAA1199 expression in duodenal adenomas could reveal its involvement in duodenal adenomatous transformation.

Recently, we reported that celecoxib & placebo, but not celecoxib & UDCA co-treatment, reduced duodenal polyp density in patients with FAP [24]. In that study, cell proliferation, apoptosis and COX-2 expression in the normal duodenal mucosa of the patients with FAP were assessed immunohistochemically, but no effects of celecoxib or celecoxib & UDCA treatment were found. Here, we quantified mRNA levels of several potential risk markers, but again, no consistent effects of either of the two interventions on levels of cell cycle related markers were observed. We did observe a significant difference in change in E-cadherin mRNA levels between both treatment groups, but pre vs. post treatment differences in E-cadherin mRNA levels were not significantly different within either of the two treatment groups. Group comparisons with larger study samples are necessary to elucidate whether actual differences in potential markers do exist.

The present study has several strengths. First, the study population of patients with FAP consists of a relatively large and unique sample from 5 out of 8 Dutch academic medical centers. Second, a new and relatively simple technique, which is not based on quantitative polymerase chain reaction (qPCR), is used to measure mRNA levels of several potential risk markers of interest simultaneously, in normal duodenal mucosa of patients with FAP and non-FAP patient controls. The following limitations are noted. First, the number of non-FAP patient controls included is relatively small. Consequently, group comparisons may lack sufficient statistical power to reveal actual differences, and comparisons in which no statistically significant difference was observed are therefore to be interpreted with caution. Second, mRNA level of each sample was determined as single measurement, however, mean mRNA levels of two different duodenal biopsies from the same patient taken at predefined locations of the duodenum were used in the analyses. Third, although of great interest, we were not able to assess mRNA expression levels in duodenal adenoma biopsy samples. No biopsy samples of adenomas were taken, as pre-intervention sampling of adenomas would have introduced bias in the primary outcome of the intervention study [24].

In summary, mRNA levels of nine potential risk parameters for malignant transformation were assessed in normal duodenal mucosa of patients with FAP and non-FAP patient controls. Markers for protection against toxins and carcinogens (GSTA1) and apoptosis (caspase-3) were lower in patients with FAP, which could contribute to the increased susceptibility for malignant transformation of normal duodenal mucosa of patients with FAP. None of the nine evaluated potential risk markers seem to be consistently effected by either celecoxib mono-treatment or celecoxib & UDCA co-treatment. COX-2 levels in normal duodenal mucosa of patients with FAP, measured at the mRNA level, were found to be very low, contrasting previous reports of immunohistochemical findings.

### Clinical perspective

Chemoprevention would be desirable to avoid duodenectomy in patients with FAP. Identification of risk markers in normal duodenal mucosa could help identify patients at increased risk for malignant transformation. Messenger RNA (mRNA) levels of nine potential risk markers were evaluated. mRNA of glutathione S-transferase A1 (GSTA1) and caspase-3 were significantly lower in patients with FAP vs. non-FAP patient controls. Therefore, protection against toxins and carcinogens (GSTA1) and apoptosis (caspase-3) seems lower in patients with FAP, which could contribute to increased susceptibility for malignant transformation of duodenal mucosa. None of the potential risk markers was consistently influenced by either celecoxib or celecoxib & UDCA.

## Conclusion

Protection against toxins (glutathione S-transferase A1) and apoptosis (caspase-3) seems lower in normal duodenal mucosa of patients with FAP, as compared to in patient controls, which could contribute to the formation of neoplasias in the duodenum of patients with FAP.

## Abbreviations

AMC: Academic Medical Centre Amsterdam; APC: Adenomatous polyposis coli; β2M: Beta-2-microglobulin; COX-2: Cyclooxygenase-2; EMC: Erasmus Medical Centre Rotterdam; FAP: Familial adenomatous polyposis; GSTA1: Glutathione S-transferase A1; GSTP1: Glutathione S-transferase P1; GSTs: Glutathione S-transferases; lef: Lymphoid enhancer factor family; LUMC: Leiden University Medical Centre; NSAIDs: Non-steroidal anti-inflammatory drugs; PGE2: Prostaglandin E2; PPARδ: Peroxisome proliferator-activated receptor δ; qPCR: Quantitative polymerase chain reaction; RUNMC: Radboud University Nijmegen Medical Centre; Tcf: T cell factor; UDCA: Ursodeoxycholic acid; UMCG: University Medical Centre Groningen.

## Competing interests

The authors declare that they have no competing interests.

## Authors’ contributions

BWHH: study design and protocol writing, patient recruitment, conducting the study protocol, data collection, analysis, and interpreting, drafting the manuscript; HMJR: data collection and analysis, drafting the manuscript; RHMM: data collection and analysis, drafting the manuscript; FMN: study design and protocol writing, conducting the study protocol, data collection and interpreting, critical review of the manuscript; WHMP: study design and protocol writing, data collection and interpreting, critical review of the manuscript. All authors read and approved the final manuscript.
